# Nasal patency and otorhinolaryngologic-orofacial features in children^☆^

**DOI:** 10.1016/j.bjorl.2017.10.014

**Published:** 2017-11-21

**Authors:** Jovana de Moura Milanesi, Luana Cristina Berwig, Luiz Henrique Schuch, Rodrigo Agne Ritzel, Ana Maria Toniolo da Silva, Eliane Castilhos Rodrigues Corrêa

**Affiliations:** a Universidade Federal de Santa Maria, Programa de Distúrbios de Comunicação Humana, Santa Maria, RS, Brazil; b Hospital Universitário de Santa Maria, Santa Maria, RS, Brazil; c Universidade Federal de Santa Maria, Departamento de Fonoaudiologia, Santa Maria, RS, Brazil; d Universidade Federal de Santa Maria, Departamento de Fisioterapia, Santa Maria, RS, Brazil

**Keywords:** Nasal obstruction, Rhinitis, Mouth breathing, Stomatognathic diseases, Mastication, Obstrução nasal, Rinite, Respiração oral, Doenças estomatognáticas, Mastigação

## Abstract

**Introduction:**

Nasal obstruction is a common symptom in childhood, related to rhinitis and pharyngeal tonsil hypertrophy. In the presence of nasal obstruction, nasal patency may be reduced, and nasal breathing is replaced by mouth breathing. Orofacial and otorhinolaryngologic changes are related to this breathing mode. Objective evaluation of upper airways may be obtained through nasal patency measurement.

**Objective:**

To compare nasal patency and otorhinolaryngologic-orofacial features in children.

**Methods:**

One hundred and twenty three children, 6–12 year-old, and of both sexes underwent speech therapy evaluation, according to Orofacial Myofunctional Evaluation protocol, clinical and endoscopic otorhinolaryngologic examination and nasal patency measurement, using the absolute and predicted (%) peak nasal inspiratory flow values.

**Results:**

Lower values of absolute and estimated peak nasal inspiratory flow values were found in children with restless sleep (*p* = 0.006 and *p* = 0.002), nasal obstruction report (*p* = 0.027 and *p* = 0.023), runny nose (*p* = 0.004 and *p* = 0.012), unsystematic lip closure during mastication (*p* = 0.040 and *p* = 0.026), masticatory speed reduced (*p* = 0.006 and *p* = 0.008) and altered solid food swallowing (*p* = 0.006 and *p* = 0.001). Absolute peak nasal inspiratory flow was lower in children with pale inferior turbinate (*p* = 0.040), reduced hard palate width (*p* = 0.037) and altered speech (*p* = 0.004). Higher absolute values were found in children with increased tongue width (*p* = 0.027) and, higher absolute and predicted (%) in children with mild everted lip (*p* = 0.008 and *p* = 0.000).

**Conclusions:**

Nasal patency was lower in children with restless sleep, rhinitis signs and symptoms, hard palate width reduced and with changes in mastication, deglutition and speech functions. It is also emphasized that most of the children presented signs and symptom of allergic rhinitis.

## Resumo

**Introdução:**

A obstrução nasal é um sintoma comum na infância relacionado a rinite e hipertrofia das tonsilas faríngeas. Na presença de obstrução nasal, a patência nasal pode estar reduzida e a respiração nasal ser substituída por respiração oral. Alterações orofaciais e otorrinolaringológicas estão relacionadas a esse modo de respiração. A avaliação objetiva das vias aéreas superiores pode ser obtida através da medida da patência nasal.

**Objetivo:**

Comparar a patência nasal e características otorrinolaringológicas e orofaciais em crianças.

**Método:**

Foram submetidas 123 crianças de seis a 12 anos, de ambos os sexos, a avaliação fonoaudiológica, de acordo com o protocolo de avaliação Miofuncional Orofacial, exame clínico e endoscópico otorrinolaringológico e medição da patência nasal, com o uso do pico de fluxo inspiratório nasal em valores absolutos e valores estimados (% pico de fluxo inspiratório nasal).

**Resultados:**

Valores mais baixos de pico de fluxo inspiratório nasal e % pico de fluxo inspiratório nasal foram encontrados em crianças com sono agitado (*p* = 0,006 e *p* = 0,002), relato de obstrução nasal (*p* = 0,027 e *p* = 0,023), rinorreia (*p* = 0,004 e *p* = 0,012), fechamento não-sistemático dos lábios durante a mastigação (*p* = 0,040 e *p* = 0,026), velocidade mastigatória reduzida (*p* = 0,006 e *p* = 0,008) e alteração da ingestão de alimentos sólidos (*p* = 0,006 e *p* = 0,001). O pico de fluxo inspiratório nasal foi menor em crianças com conchas inferiores pálidas (*p* = 0,040), redução da largura do palato duro (*p* = 0,037) e alterações da fala (*p* = 0,004). Valores maiores foram encontrados em crianças com largura da língua aumentada (*p* = 0,027). Valores maiores de pico de fluxo inspiratório nasal e % pico de fluxo inspiratório nasal foram observados em crianças com lábio levemente evertido (*p* = 0,008 e *p* = 0,000).

**Conclusões:**

A patência nasal foi menor em crianças com sono agitado, sinais e sintomas de rinite, redução da largura do palato duro e alterações nas funções de mastigação, deglutição e fala. Enfatiza-se também que a maioria das crianças apresentava sinais e sintomas de rinite alérgica.

## Introduction

Nasal obstruction is the most common symptom in children and may be related to presence of inflammatory nasal conditions as rhinitis and pharyngeal tonsil hypertrophy.^1^, ^2^ In presence of nasal obstruction, nasal breathing is replaced by mouth breathing (MB).^3^, ^4^, ^5^ The upper airways may be evaluated through nasal patency measurement. Peak Nasal Inspiratory Flow (PNIF) is an objective, reliable and easy-to-use instrument for detection of obstructive and/or inflammatory nasal patency disorder, inclusive in children.^6^, ^7^ This instrument has been used in the nasal obstruction intensity and as a treatment result evaluation and follow-up.^1^, ^8^ Authors set reference values of PNIF for 8–15 year old Brazilian healthy children.^7^ Therefore, besides the detection of nasal patency disorder, it is possible to quantify its magnitude and relates it to Otorhinolaryngologic (OTRL) and orofacial changes.

Clinical aspects and complementary exams are used for upper airway assessments. Paroxysmal sneezing, nasal itching and obstruction, runny nose, oropharyngeal itching, ocular hyperemia and itching, hyaline secretion and inferior turbinates hypertrophy and paleness are the main signs and symptoms of rhinitis.^9^ This condition is one of the most prevalent respiratory disease in childhood.^10^ Concerning complementary exams, nasofibroendoscopy and/or cavum radiography are essential to identify MB etiological factors, mainly pharyngeal tonsil hypertrophy and its classification.^2^

MB arises, in attempt of more efficient airflow passage, causing some changes.^3^ The most common consequences of the mouth-breathing mode are half-open lip posture, hypofunction of orbicularis oris muscle, everted lower lip, tongue position in the mouth floor or interposed between the arcades, narrow and deep hard palate, atypical deglutition and alterations in craniofacial development, such as increased lower third of the face.^3^, ^11^, ^12^, ^13^ It is believed that these changes are relative to the nasal obstruction magnitude, i.e., nasal patency intensity. Besides, the influence of etiological factor on MB consequences may be diverse and need more investigation. The aim of this study was to compare nasal patency and otorhinolaryngologic-orofacial features in 6–12 year old children.

## Methods

This prospective study has derived from a project titled “Integrated characterization and evaluation of orofacial motricity and body posture diseases – phase II”, approved in Ethics and Research Committee of Universidade Federal de Santa Maria, under protocol 08105512.0.0000.5346 with observational and cross-sectional design.

For this study, 6–12 year old children of both sexes were recruited from an elementary school. All parents or tutors were informed about the procedures and signed the Consent Form, according to 466/12 resolution of National Health Committee (NHC). Children with missed or permanent dentition and normal ventilatory function, verified by spirometry, were included. Spirometric evaluation (One Flow – Clement Clarke) was carried out, according to the American Thoracic Society^14^ and *Sociedade Brasileira de Pneumologia e Tisiologia*.^15^ Some exclusion criteria were established: signs and symptoms of rhinitis exacerbation, antihistaminic or corticoid therapy oral or topic during the last 30 days, undergoing orthodontic treatment, physiotherapy or speech therapy, with facial surgery or trauma or evident signs of neurological disease and/or craniofacial malformation (including stomatognathic system alterations provided of these neurological diseases and malformations). Children with signs and symptoms of infectious rhinitis and others types of rhinitis were also excluded.

**Figure 1:**
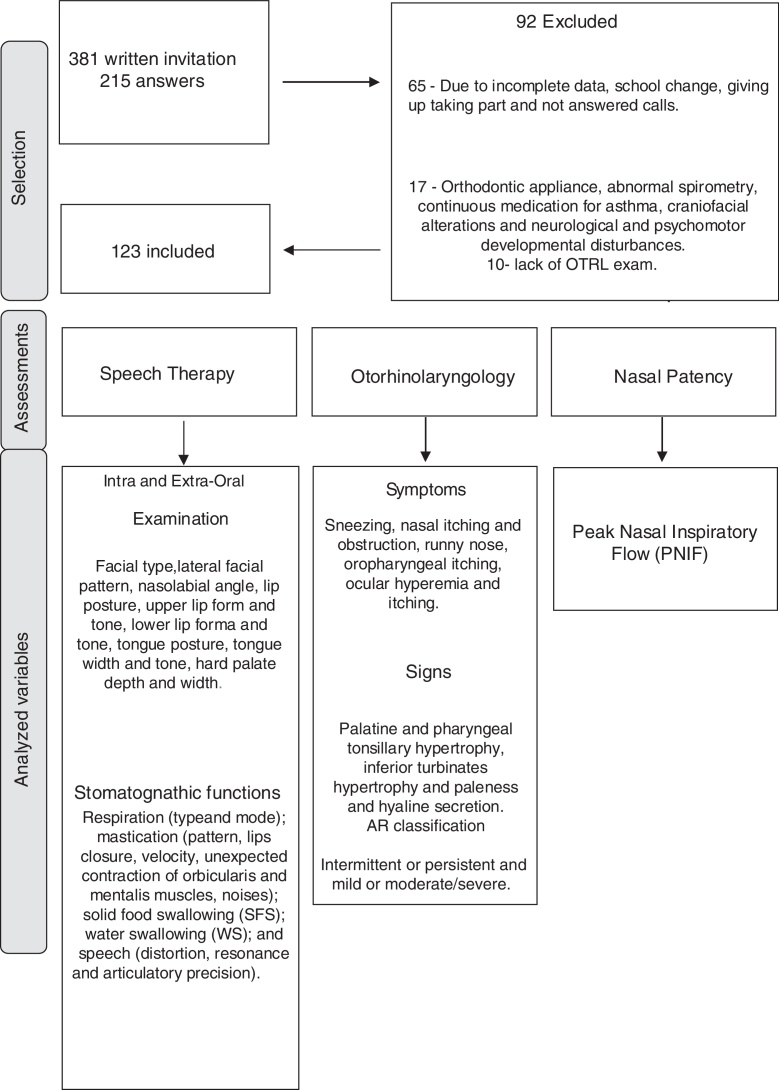
Flowchart of selection, evaluation procedures and analyzed variables.

All participants underwent speech therapy, OTRL and physical therapy assessments through evaluators with more than 5 years’ experience and blind to each other. Selection and evaluation processes are demonstrated in Fig. 1, as well as the analyzed variables.

Stomatognathic system evaluation was carried out by an experienced speech therapist in orofacial motricity by means of MBGR protocol.^16^ Masticatory, deglutition and speech functions were evaluated according to protocol instructions, Photographs and filming were carried out.

An otorhinolaryngologist evaluated the children, considering aspects such as palatine and pharyngeal tonsillary hypertrophy, nasal septum deviation and nasal mucosa edema, by means of oroscopy and anterior rhinoscopy. Nasoendoscopy or lateral cavum X-ray, depending on the child acceptance, as also carried out. Palatine and pharyngeal tonsils assessments followed, respectively, according to Brodsky and Koch^17^ and Parikh^18^ classifications. Additionally, the presence of rhinitis signs and symptoms were qualitatively analyzed, such as: inferior turbinates hypertrophy and paleness, hyaline secretion, paroxysmal sneezing, nasal itching and obstruction, runny nose, oropharyngeal itching, ocular hyperemia and itching.^9^ For AR classification, the ARIA (Allergic Rhinitis and its Impact on Asthma) initiative was used,^19^ related to the symptom frequency (intermittent or persistent) and intensity (mild or moderate/severe).

PNIF measure was used for objective nasal patency assessment. It was evaluated by a physical therapist using the In Check Inspiratory Flow Meter (Clement Clarke International, the United Kingdom), from residual volume (RV), i.e., a complete expiration followed by a nasal deep inspiration as fast and strong as possible, with mouth closed and a well-adapted mask to face. The highest value obtained from three repetitions was recorded.^20^ The obtained values were transformed in percentage of predicted values of PNIF, set by Ibiapina et al.,^7^ according to sex and stature.

The STATISTICA 9.1 software (Statistica for Windows – release 9.1 Stat Soft) was used for descriptive and inferential data analysis, considering *p* < 0.05 as significant level. Data were exposed in median and interquartile range. Lilliefors test was used for data normality analysis. Nasal patency values were compared to otorhinolaryngologic-orofacial features through Mann–Whitney, Kruskal–Wallis and Multiple Comparisons Tests.

## Results

This study analyzed nasal patency and otorhinolaryngologic-orofacial features, comparing normal and altered conditions, in 123 children, 69 boys and 54 girls, mean age of 8.5 ± 1.6 years old.

Table 1 shows the %PNIF, PNIF values (predicted and absolute) and data from anamnesis and OTRL examination. Significant lower values of PNIF and %PNIF were found in children with restless sleep, nasal obstruction report and runny nose. Significant lower PNIF in children with pale inferior turbinate was also found.

**Table 1:** PNIF (predicted and absolute values) and data from anamnesis and OTRL examination.

Variables	*n*	%PNIF	*p*	PNIF	*p*
*Restless sleep*					
No	62	92.8 (82–109)	0.006^a^	99.0 (80–112)	0.002^a^
Yes	61	82.0 (68–94)		80.0 (70–100)	

*Snoring*					
No	53	87.5 (68–103)	0.723	90.0 (70–110)	0.628
Yes	70	89.0 (77–101)		90.0 (75–100)	

*Open mouth sleep*					
No	42	88.7 (77–106)	0.510	90.0 (80–112)	0.676
Yes	81	87.6 (71–100)		90.0 (75–100)	

*Nasal obstruction*					
No	59	92.4 (80–104)	0.027^a^	92.0 (80–110)	0.023^a^
Yes	64	84.8 (67–99)		80.0 (70–100)	

*Runny nose*					
No	81	92.7 (79–107)	0.004^a^	95.0 (80–110)	0.012^a^
Yes	42	82.3 (67–92)		80.0 (65–100)	

*Nasal itching*					
No	62	92.7 (78–104)	0.074	91.0 (80–110)	0.052
Yes	61	85.0 (68–97)		82.0 (70–100)	

*Sneezing*					
No	39	93.0 (78–111)	0.165	95.0 (80–110)	0.382
Yes	84	87.3 (69–99)		90.0 (70–101)	

*Pharyngeal tonsills*^b^					
Non obstructive	46	87.5 (78–103)	0.789	91.0 (75–102)	0.928
Obstructive	28	89.9 (80–110)		95.0 (77–105)	

*Palatine tonsills*					
Non obstructive	109	87.5 (77–103)	0.490	90.0 (75–105)	0.371
Obstructive	14	90.0 (66–95)		85.0 (65–100)	

*Lower turbinate hypertrophy*					
No	61	85.0 (69–97)	0.051	82.0 (70–100)	0.171
Yes	62	92.0 (80–108)		96.5 (78–105)	

*Pale lower turbinate*					
No	30	91.4 (82–111)	0.190	100.0 (82–118)	0.040^a^
Yes	93	87.1 (71–101)		85.0 (70–100)	

*Hyaline secretion*					
No	42	89.3 (76–101)	0.940	95.0 (78–105)	0.530
Yes	81	87.5 (71–103)		90.0 (70–100)	

*Rhinitis frequency*					
No	16	83.8 (70–100)	0.866	88.5 (72–110)	0.689
Intermittent	56	89.7 (73–101)		85.0 (72–100)	
Persistent	51	87.5 (78–104)		98.0 (75–102)	

*Rhinitis intensity*					
No	16	83.8 (70–100)	0.244	88.5 (72–110)	0.692
Mild	60	90.8 (77–108)		90.0 (79–110)	
Moderate/severe	47	87.1 (65–97)		90.0 (70–100)	

Values expressed in median and interquartile range.

% PNIF, Predicted Peak Nasal Inspiratory Flow; PNIF, Peak Nasal Inspiratory Flow (L/min); OTRL, Otorhinolaryngologic.

a Kruskal–Wallis ANOVA; Mann–Whitney Test; *p*<0.05.

b Missing Data: 49.

In Table 2 PNIF and %PNIF values are described, comparing them to stomatognathic variables related to structures evaluated by MBGR protocol, with significant difference in lower lip form, tongue and hard palate width.

**Table 2:** PNIF (predicted and absolute values) and stomatognathic structures (MBGR protocol).

Variables	*n*	%PNIF	*p*	PNIF	*p*
Facial type					
Medium	57	87.1 (74–100)	0.730	85 (70–110)	0.254
Long	27	90 (77–101)		95.7 (80–110)	
Short	39	87.4 (68–107)		86.0 (70–100)	

*Lateral facial pattern*					
Straight	58	86.9 (68–101)	0.364	82 (70–100)	0.227
Convex	61	90.5 (79–101)		95 (80–102)	
Concave	4	93.2 (75–108)		100 (75–122)	

*Nasolabial angle*					
Around 90°–110°	82	87.3 (71–104)	0.971	90.0 (70–110)	0.769
Acute (<90°)	16	90.3 (65–106)		91 (67–106)	
Obtuse (>110°)	25	87.6 (84–94)		95 (80–100)	

*Lip Posture*					
Close	71	90.5 (77–107)	0.345	90.0 (78–110)	0.637
Close with tension	13	87.5 (80–100)		90.0 (80–100)	
Half open/open	39	86.4 (63–101)		95.0 (60–100)	

*Upper lip form*					
Normal	88	87.3 (71–101)	0.408	85 (70–100)	0.093
Gull wing	35	90 (77–106)		100 (80–110)	

*Upper lip tonus*					
Normal	86	89.3 (78–101)	0.463	90 (75–102)	0.474
Reduced	36	86.1 (64–110)		93.5 (72–105)	
Increased	1	67.8 (–)		65 (–)	

*Lower lip form*					
Normal	52	82 (69–93)^a^	0.008^a^	80 (70–90)^b^	0.000^a^
Mild everted	60	93 (78–112)^b^		100 (80–115)^b^	
Everted	11	92 (85–100)		100 (80–102)	

*Lower lip tonus*					
Normal	69	90.5 (77–103)	0.514	90 (75–110)	0.938
Reduced	54	87.1 (68–101)		96.5 (75–100)	
Increased	0	–		–	

*Tongue posture*					
Not visible	86	90.2 (79–104)	0.131	90.0 (78–110)	0.301
In the mouth floor	22	86.1 (53–97)		90.0 (60–100)	
Between the teeth	15	82.0 (65–107)		80.0 (60–110)	

*Tongue width*					
Normal	89	86 (70–101)	0.075	85 (70–100)^b^	0.027^a^
Reduced	1	125.5 (–)		130 (–)	
Increased	33	92 (81–103)		98 (80–120)^b^	

*Tongue tonus*					
Normal	66	90.7 (77–104)	0.270	95 (75–110)	0.276
Reduced	57	85.9 (69–101)		82.0 (70–100)	
Increased	0	–		–	

*Hard palate depth*					
Adequate	52	86.9 (72–06)	0.962	90 (70–105)	0.750
Reduced (shallow)	2	90.1 (67–112)		82.5 (65–100)	
Increased (deep)	69	89.3 (78–101)		90.0 (80–102)	

*Hard palate width*					
Adequate	77	90.7 (79–107)	0.080	95.0 (80–100)^b^	0.037^a^
Increased (wide)	2	90.1 (67–112)		82.5 (65–100)	
Reduced (narrow)	44	84.5 (67–96)		80 (67–100)^b^	

Values expressed in median and interquartile range.

%PNIF, Predicted Peak Nasal Inspiratory Flow; PNIF, Peak Nasal Inspiratory Flow (L/min); MBGR, Marchesan, Berretin-Felix, Genaro, Rheder.

a Kruskal–Wallis ANOVA; Mann–Whitney Test; *p* < 0.05.

b Categories with statistic difference.

Comparison between PNIF and %PNIF values with variables related to stomatognathic functions, evaluated by MBGR protocol, is shown in Table 3. Significantly different values were found in mastication, solid food swallowing and speech functions.

**Table 3:** PNIF (predicted and absolute values) and stomatognathic functions (MBGR protocol).

Variables	*n*	%PNIF	*p*	PNIF	*p*
*Respiratory mode*					
Nasal	53	87.8 (71–103)	0.699	88.9 (70–110)	0.646
Oral	70	89.0 (78–101)		95.0 (75–100)	

*Masticatory pattern*					
Bilateral alternate/unilateral preferential	98	89.0 (74–101)	0.838	90.0 (70–110)	0.932
Unilateral chronic/bilateral simultaneous	25	87.1 (76–103)		90.0 (78–100)	

*Lip closure on mastication*					
Systematic	90	91.0 (77–106)^b^	0.040^a^	93.5 (78–110)^b^	0.026^a^
Unsystematic	30	85.0 (68–95)^b^		80.0 (65–100)^b^	
Absent	3	67.0 (51–85)		65.0 (55–80)	

*Masticatory speed*					
Normal	88	87.5 (77–102)^b^	0.021^a^	90.0 (76–101)^b^	0.016^a^
Increased	30	96.1 (78–103)^b^	0.006^a^	99.0 (75–110)^b^	0.008^a^
Reduced	5	53 (51–74)^b^		55.0 (50–70)^b^	

*Solid food swallowing (SFS)*					
Normal	108	91.0 (77–106)	0.006^a^	95.0 (78–110)	0.001^a^
Altered	15	82.0 (67–87)		80.0 (65–80)	

*Tongue posture (SFS)*					
Not seen	104	90.2 (75–105)	0.063	91.0 (75–110)^b^	0.023^a^
Behind the teeth	4	92.0 (86–103)		98.0 (89–104)	
Between teeth/interdental	15	84.0 (51–87)		80.0 (55–90)^b^	

*Water swallowing*					
Normal	83	89.4 (78–103)	0.172	90 (80–110)	0.097
Altered	40	86.5 (66–102)		80.0 (70–100)	

*Tongue posture (WS)*					
Not seen	22	91.3 (63–101)	0.268	92.5 (70–110)	0.171
Behind the teeth	61	87.6 (80–104)		90.0 (80–110)	
Between teeth/interdental	40	86.5 (66–102)		80.0 (70–100)	

*Speech*					
Normal	76	89.3 (80–102)	0.092	98.0 (80–110)	0.004^a^
Altered	47	82.6 (67–103)		80.0 (65–100)	

Values expressed in median and interquartile range.

%PNIF, Predicted Peak Nasal Inspiratory Flow; PNIF, Peak Nasal Inspiratory Flow (L/min); MBGR, Marchesan, Berretin-Felix, Genaro, Rheder.

a Kruskal–Wallis ANOVA; Mann–Whitney Test; *p* < 0.05.

b Categories with statistic difference.

## Discussion

The literature is not quite clear in relation to the use of objective tools that are able to quantify nasal patency in children. Rhinomanometry, accoustic rhinometry and some more sophisticated image exams are methods for nasal function assessment. Noninvasive and easy to use evaluation procedures have remained a constant challenge for clinical practice. Currently, PNIF has been used in upper airway assessment,^8^, ^21^, ^22^ but in the reviewed literature, studies about nasal patency, related to rhinitis signs and symptoms as well as orofacial aspects in children, have not been found.

Reduced values of %PNIF and PNIF were found in children with restless sleep, nasal obstruction report and inferior nasal turbinate paleness. Decreased nasal patency may suggest that there are some problems to breathing and this is reflected in sleep. One study, using cephalometric analysis, found a reduced airway pharyngeal space in children with high risk for sleep disorder, compared to low risk.^23^ Forty-three percent of sleep-disordered breathing symptoms were found in 65 symptomatic children with nasal obstruction.^24^

This sample shows that children with nasal obstruction report presented reduction of 7.6 L/min in the PNIF value. These results demonstrate an association between symptomic and the objective measure of nasal patency. Nasal obstruction is the main symptom of rhinitis and it may be attributed to nasal mucosa inflammation and increased secretion.^8^ PNIF has been strongly associated with rhinitis, diagnosed through anterior rhinoscopy.^25^ Furthermore, authors have demonstrated good correlation between PNIF and clinical scores of nasal obstruction.^8^, ^20^, ^26^ It must be emphasized that only clinical evaluation may be insufficient at detecting nasal obstruction, once the obstruction detected through objective examination may be different that the one reported by children, i.e., their perception may be underestimated or overestimated.^8^, ^24^, ^26^ Additionally, information provided by PNIF is different from qualitative symptom reports.^25^ Therefore, a combination of objective and subjective methods of nasal patency assessments is suggested.^20^, ^25^, ^27^

Rhinitis subjects are prone to present reduced PNIF values.^8^ In a recent study, rhinitis children have presented, respectively, %PNIF mean values of 64.1% and 90.7% before and after treatment.^1^ In the present study, PNIF and %PNIF values in children with nasal signs and symptoms were respectively, 80 L/min and 80%. Healthy Brazilian children, 8–15 years old, showed PNIF absolute values of 111.6 L/min in boys and 99.2 L/min in girls.^7^ It was observed that children in this study presented similar values to healthy children.

Consequences of MB mode have been widely studied for the last several years.^4^, ^28^, ^29^, ^30^, ^31^ Although, specific consequences of decreased nasal patency still remain inconsistent. Results of stomatognathic system structures were rather varied. Unexpectedly, higher PNIF values were found in children with mild eversion lower lip and increased tongue width. Decreased PNIF values were found in children with narrower palate. There were no differences in PNIF values related to stomatognatic system structures.

Absence of lip sealing and interposition of tongue between teeth and hard palate atresia were found in children with adenotonsillar hypertrophy.^30^ Meanwhile, these changes that characterize MB may not be related to increased nasal resistance or reduced nasal airflow,^32^ but they may be due to oral habits.

In the present study, children with or without changes in orofacial structures presented similar PNIF values to healthy children.^7^ Such findings may be explained considering MB as a consequence of oral habits^10^, ^33^ or transient edema of nasal mucosa.^34^

Concerning stomatognatic functions, %PNIF and PNIF values were statistically lower in children with unsystematic lip closure during mastication and reduced masticatory speed. Such values of %PNIF and PNIF were respectively, 53% and 55 L/min, lower than the ones found in healthy children.^7^ A study has detected MB mode and changes in masticatory and swallowing functions in 30 allergic rhinitis children.^13^ Authors have also found significant correlations between increase of nasal obstruction signs and symptoms scores and the presence of masticatory and swallowing dysfunctions.

Coordination between breathing and mastication is a complex process and as breathing is a more vital function, masticatory movement may be interrupted during MB.^35^ In the present study, unsystematic lip closure was found, as well as in another study, with children presenting adenotonsillary hypertrophy.^4^ Furthermore, mentalis and orbicularis oris tension and tongue interposition between teeth during swallowing may occur, as a compensatory mechanism necessary to keep food inside the mouth.^4^

Reduced %PNIF and PNIF values were also observed in children with solid deglutition altered. Smaller pharyngeal airway space, detected by means of teleradiographs in 7–11 years old children with atypical deglutition was found, compared to a control group and considering age and sex variables.^36^

Children with speech disorders presented significant lower PNIF values than the ones with normal speech function. The most common speech disorders described in MB are: forward tongue during lingual dental phonemes; imprecision in bilabial and fricative phonemes; and frontal and lateral lisp.^37^ Lower mandibular movement speed during speech was found in rhinitis children, compared to a control group, but with no statistical significance.^38^ According to the authors, nasal obstruction would be related to mobility, tonus and posture alteration of phono-articulatory organs.

It seems that the stomatognatic functions are firstly changed and this could cause structural alterations over time. The changes associated with decreased nasal patency were those that are also associated with MB. This reinforces the need to treat nasal obstruction along with treatment of stomatognatic functions. However, the fact that the sample presented a large number of children with signs and symptoms of allergic rhinitis associated with decreased nasal patency, dictates that the results should be considered with caution.

Despite the fact that orofacial evaluation has an observational and qualitative nature, some systematic and standardized protocols have been used, allowing the comparison among studies.^16^, ^30^ Another fact being considered refers to the volitional character of PNIF exam, mainly with children. Therefore, it is suggested that further research with quantitative analysis in the orofacial motricity and concerning methodological aspects of PNIF test, be considered.

It is relevant to consider that nasal obstruction may cause structural and functional stomagnathic changes, in order to compensate the airflow impairment, given that lower patency was found in children with masticatory and deglutition dysfunction. In order not to be neglected, these functions demand more attention, since they are neither easily realized by children nor observed by parents and professionals.

## Conclusion

Nasal patency was lower in children with restless sleep, rhinitis signs and symptoms, reduced hard palate width and changes in mastication, deglutition and speech functions. It is also emphasized that most of the children presented signs and symptom of allergic rhinitis.

## Conflicts of interest

The authors declare no conflicts of interest.
